# Invasion History of the Oriental Fruit Fly, *Bactrocera dorsalis*, in the Pacific-Asia Region: Two Main Invasion Routes

**DOI:** 10.1371/journal.pone.0036176

**Published:** 2012-05-02

**Authors:** Xuanwu Wan, Yinghong Liu, Bin Zhang

**Affiliations:** Chongqing Key Laboratory of Entomology and Pest Control Engineering, College of Plant Protection, Southwest University, Chongqing, China; American Museum of Natural History, United States of America

## Abstract

The oriental fruit fly, *Bactrocera dorsalis*, was initially recorded in Taiwan Island in 1912, and has dispersed to many areas in the Pacific-Asia region over the last century. The area of origin of the species may be confidently placed in South-East China. However, routes of range expansion to new areas and underlying population processes remain partially unclear, despite having been the subject of several studies. To explore the invasion history of this species, a partition of the *cox1* gene of mitochondrial DNA was used to investigate genetic diversity, haplotype phylogeny and demographic history of 35 populations, covering China and South-East Asia and including marginal populations from Pakistan and Hawaii. Based on neighbor-joining tree analysis and the distribution of haplotypes, two main invasion routes are inferred: one from South-East China to Central China, another from South-East China to South-East Asia, with both routes probably coinciding in Central China. Populations in Taiwan Island and Hainan Island might have originated in South-East China. The marginal populations in Pakistan and Hawaii might have undergone founding events or genetic bottlenecks. Possible strategies for the control of this species are proposed based on the invasion history and reconstructed expansion routes.

## Introduction

Biological invasions constitute a growing threat to human economic activities and health, agriculture, and natural environments. Although many species of plants, animals and other organisms have been introduced around the world by human activities [1], invasion processes have been relatively slow in previous centuries. However, the pace has been accelerated by globalization [2]. Currently, human-mediated species invasions are a significant component of global environmental change [3].

An understanding of the history of invasion processes, i.e. a description of geographical pathways of invading populations, provides useful information about the origin and genetic composition of such populations and can be highly advantageous in attempts to quarantine, control or eradicate an invading species. Investigating the genetic architecture of invading populations offers an opportunity to infer a species' invasion history using molecular techniques [4]. Several different invasion scenarios have been identified, such as independent introductions [5], multiple introductions [6], [7], population-genetic bottlenecks [8], founder events [9], invasive bridgeheads [10]–[12] and genetic admixture [13]–[15].

Although the reliability of some traits of mitochondrial DNA (mtDNA), such as neutrality and constant mutation rate, has been criticized [16]–[20], mtDNA markers are universally used in historical phylogeography. In contrast to nuclear markers, mtDNA exhibits some special evolutionary traits that are useful in the study of invading populations: the property of nonrecombination enables a relatively precise retracing of the origins of invasive populations [21]; high copy numbers in cells facilitate the amplification of DNA sequences, especially when historical specimens are used [22]; less effective gene size than in nuclear DNA, resulting from uni-parental inheritance, make it sensitive to selective neutrality and the loss of mutation-drift equilibrium and male-to-female sex ratio balance [22]. These characteristics combine to make mtDNA markers a mainstay of phylogeography [23].

The oriental fruit fly *Bactrocera dorsalis*, a phytophagous species of the Tephritidae family, was first recorded in Taiwan Island, China, in 1912 [24]. Enormous damage to agricultural production has been caused by this species through its larvae, which feed on fruit. Negative impacts on biodiversity in invaded regions have also been observed [25], [26]. Due to the species' broad host range, wide climate tolerance and high dispersal capacity [27], its distribution range has covered the Asia-Pacific region in the last century, ranging from India to Hawaii and encompassing all of South-East Asia. Although the oriental fruit fly has been successfully eradicated in several regions (Ryukyu Islands in Japan, Nauru, Guam and Northern Mariana Islands) [28], the invasion process is rapid and continuous, and an invasion trend towards the poles in the wake of global environment changes has been predicted [28]. Despite the current and potential risk posed by this fruit fly, information about the invasion process of this species is scarce. Previous studies by Aketarawang et al. (2007) [29] and the authors [30] supported the supposition that the oriental fruit fly may have originated in the South-East China region facing the South China Sea, and spread further inland from there.

In this study, we aim to a) expand our previous work on oriental fruit fly expansion to cover all areas of the species' worldwide distribution, b) reconstruct the routes of the species' expansion from its origin in South-East China to areas of recent colonization in South-East Asia and to marginal populations in Pakistan and Hawaii, and c) discuss the application of these results to the planning of appropriate control measures.

## Materials and Methods

### Sampling, DNA extraction and amplification

A total of 552 oriental fruit flies from 35 populations were used in the analysis, including 256 *B. dorsalis* adults collected from 14 locations in China and one in Pakistan in 2008–2010 (for sampling information see Table 1). No specific permissions were required for these locations/activities, the locations are not privately owned or protected in any way. Information from these locations was completed with 296 additional sequences obtained from GenBank that together cover 20 locations in eight countries and constitute a reasonably complete coverage of the distributional range of the species (for sequence accession numbers see Table S1).

DNA was extracted using DNeasy Blood and Tissue Kits (QIAGEN) on each individual fly. A 505 base-pair partial segment of the mtDNA *cox1* gene was amplified following Shi et al. (2005) [31]. PCR products were purified and sequenced on both strands by Invitrogen Biotechnology Co. (Shanghai, China). Unique sequences were deposited in GenBank under accession number JN643923-JN644053.

**Table 1:** Sampling information.

Location	ID	Number	Longitude	Latitude	Year
Fuzhou, China	FZ	17	119°28′(E)	26°15′(N)	2009
Xiamen, China	XM	15	118°05′(E)	24°28′(N)	2005
*Quanzhou, China*	QZ	13	118°43′(E)	25°29′(N)	-
Zhaoqing, China	ZQ	22	112°27′(E)	23°02′(N)	2005
Maoming, China	MM	5	110°87′(E)	22°26′(N)	-
Shaoguan, China	SG	5	114°15′(E)	24°17′(N)	-
Guangzhou, China	GZ	16	113°28′(E)	23°18′(N)	2009
Pingxing, China	PX	13	106°45′(E)	26°06′(N)	2005
Nanning, China	NN	20	108°46′(E)	22°78′(N)	2010
Huaxi, China	HX	15	106°67′(E)	26°44′(N)	2009
Bawangling, China	BWL	16	109°03′(E)	19°06′(N)	2007
Wenchang, China	WC	20	110°76′(E)	19°68′(N)	2010
Wuhan, China	WH	20	114°36′(E)	30°48′(N)	2009
Nanchang, China	NC	20	115°79′(E)	28°62′(N)	2009
Jianshui, China	JS	20	102°82′(E)	23°70′(N)	2010
Hekou, China	HK	8	103°88′(E)	22°59′(N)	2009
Jinghong, China	JH	21	100°48′(E)	21°59′(N)	2005
Ruili, China	RL	25	97°51′(E)	24°01′(N)	2006
Panzhihua, China	PZH	14	101°72′(E)	26°58′(N)	2008
Jiangjin, China	JJ	20	106°25′(E)	29°08′(N)	2009
Wanzhou, China	WZ	20	108°50′(E)	30°75′(N)	2009
Wulong, China	WL	20	108°97′(E)	28°42′(N)	2009
Xiushan, China	XS	20	107°02′(E)	29°30′(N)	2009
*Qingpu, China*	QP	16	121°14′(E)	31°34′(N)	-
*Taiwan, China*	TW	12	121°55′(E)	24°95′(N)	-
Yei Bai, Vietnam	YB	21	104°86′(E)	21°70′(N)	2006
Muang Khu, Laos	MK	20	102°50′(E)	21°08′(N)	2006
Louangphabang, Laos	LOU	10	102°35′(E)	20°06′(N)	2008
Mandalay, Myanmar	MAN	19	96°03′(E)	21°59′(N)	2005
Bhamo, Myanmar	BHA	28	97°17′(E)	24°16′(N)	2006
*Thailand*	THA	10	101°09′(E)	16°97′(N)	-
*Phom Penh, Cambodia*	PP	5	104°94′(E)	11°65′(N)	-
Lahore, Pakistan	LAH	16	74°35′(E)	31°54′(N)	2010
Himachal Pradesh, India	HP	5	76°32′(E)	32°06′(N)	2009
*Honolulu, USA*	HON	20	157°81′(W)	21°32′(N)	-

Populations in italics are shown with geographical coordinates that have been estimated to our best knowledge using the information provided in the original publication/GenBank record.

### Data analysis

Sequences were aligned using ClustalX 2.0 [32] and then corrected manually. ARLEQUIN 3.5 [33] was used to identify unique haplotypes. Descriptive statistics (nucleotide diversity, number of haplotypes, number of variable sites, average number of nucleotide differences and haplotype diversity) were calculated with DNAsp 5.0 [34].

The Kimura two-parameter model in MEGA 5.0 [35] was used to estimate pairwise genetic distances of the 35 populations, then the population phylogenetic tree was reconstructed using the neighbor-joining (NJ) method in PHYLIP 3.69 [36], which is the most widely used method for building phylogenetic trees from distances [37]. Median-joining (MJ) networks of haplotypes were constructed using NETWORK 4.6 to infer the evolutionary relationships of haplotypes [38], [39].

**Figure 1:**
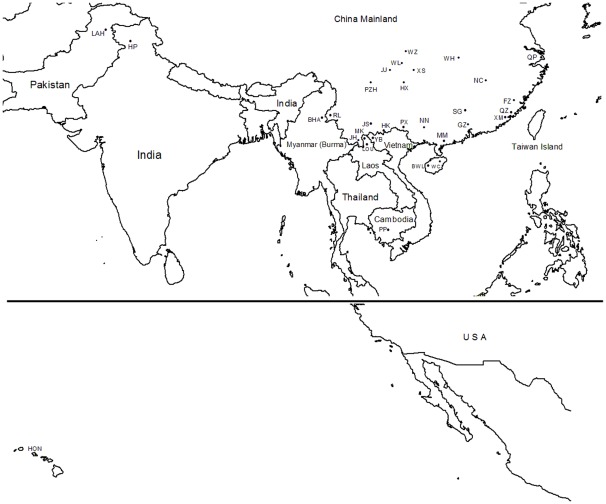
Collection sites. See Tab. 1 for complete collection information.

To infer asymmetric immigration rates between different regions, seven regions were defined based on geographic location and previous studies [30], [31] (Fig. 1). Definitions were as follows (population codes in parentheses): 1) Southeast China (XM, QZ, FZ SG, MM, ZQ and GZ); 2) Taiwan Island (TW); 3) Hainan Island (WC and BWL); 4) Central China (QP, NN, HX, NC, WH, JJ, WZ, WL, XS, JS and PZH); 5) Southeast Asia (PX, JH, HK, RL, YB, PP, THA, LOU, MK, MAN and BHA); 6) South Asia ( LAH and HP); 7) Hawaii (HON).

MIGRATE 3.27 [40] was used to estimate mutation-scaled effective immigration rate for entering and leaving each region per generation (*M* = *m*/*μ*, where *m* is immigration rate and *μ* is mutation rate per site per generation), and mutation-scaled effective population sizes (*Θ* = *N_e_μ*, where *N_e_* is effective population size), by applying a Bayesian search strategy. Four independent MIGRATE runs of 20,000,000 generations with different random start seeds were performed to examine the consistency of the results, with the first 10,000 generations discarded as “burn-in”. Analysis of molecular variation (AMOVA) and computation of fixation indices (F_ST_) between pairwise regions were implemented in ARLEQUIN.

The demographic history of each region and of all populations pooled together was examined using mismatch distributions, population size before expansion (*θ_0_*), population size after expansion (*θ_1_*), population expansion time (*τ*), Tajima's D, Fu's *FS*, and sum of squared deviations (SSD) between observed and expected mismatches. All parameters were calculated using ARLEQUIN and tested against the expected values of a recent population expansion with 1000 bootstrap replicates.

## Results

### Genetic diversity

A total of 217 unique haplotypes were identified. Of these, 51 haplotypes were shared by at least two populations, the most frequent haplotype H3 being present in 16 populations. The HON population did not share any haplotypes with other populations. Detailed information of each population's haplotype composition is shown in Table S2.

Basic descriptive indices of genetic diversity for each population are shown in Table 2. The number of variable sites (V) ranged from 4–29. Haplotype diversity (H) ranged from 0.3250–1.0000, nucleotide diversity (π) from 0.0026–0.0128, and average number of nucleotide differences (k) from 1.3000–6.5000. Almost all populations showed high levels of genetic diversity, except for the LAH and HON populations.

**Table 2:** Genetic diversity indices.

Population	V	N	H	π	k
FZ	13	9	0.8824	0.0076	3.8529
XM	15	12	0.9714	0.0091	4.5905
QZ	12	9	0.9103	0.0070	3.5128
ZQ	12	7	1.0000	0.0081	4.0952
MM	10	5	1.0000	0.0087	4.4000
SG	11	5	1.0000	0.0088	4.4000
GZ	19	12	0.9417	0.0081	4.0750
PX	15	11	0.9615	0.0065	3.2564
NN	25	19	0.9947	0.0094	4.7211
HX	16	8	0.7333	0.0066	3.3524
BWL	19	16	1.0000	0.0079	3.9833
WC	24	15	0.9684	0.0087	4.3947
WH	20	12	0.9000	0.0075	3.7947
NC	22	12	0.9263	0.0083	4.1895
JS	27	18	0.9895	0.0092	4.6263
HK	13	8	1.0000	0.0088	4.4286
JH	10	8	0.9048	0.0075	3.8095
RL	16	8	0.8467	0.0090	4.5267
PZH	13	10	0.9231	0.0052	2.6484
JJ	27	18	0.9895	0.0081	4.0895
WZ	28	16	0.9684	0.0093	4.7000
WL	29	19	0.9947	0.0106	5.3263
XS	27	16	0.9790	0.0107	5.3842
QP	11	6	0.8167	0.0069	3.4750
TW	15	8	0.8939	0.0089	4.4849
YB	14	5	0.7667	0.0092	4.6381
MK	9	5	0.7526	0.0070	3.5368
LOU	23	10	1.0000	0.0120	6.0444
MAN	23	17	0.9883	0.0095	4.8070
BHA	19	11	0.8413	0.0069	3.4735
THA	14	8	0.9556	0.0089	4.5111
PP	9	4	0.9000	0.0095	4.8000
LAH	4	2	0.3250	0.0026	1.3000
HP	15	5	1.0000	0.0128	6.5000
HON	8	5	0.6000	0.0034	1.7000

V, number of variable sites; n, number of unique haplotypes; H, haplotype diversity; π, nucleotide diversity; k, average number of nucleotide differences.

### Population genetic structure and gene flow

The AMOVA analysis revealed that the largest amount of genetic differentiation (86.23%) was to be found within populations, while genetic differentiation among groups (6.41%) and among populations within each group (7.36%) was limited. All fixation indices tested as highly significant (P<0.01) (Table 3).

**Table 3:** Partitioning of genetic variation at different hierarchical levels.

Source of variation	d.f.	Sum of squares	Variance components	Percentage of variation	Fixation indices
Among groups	6	89.823	0.15104 Va	6.41	F_CT_ = 0.07867**
Among populations within groups	28	132.723	0.17359Vb	7.36	F_SC_ = 0.13770**
Within populations	517	1051.008	2.03290Vc	86.23	F_ST_ = 0.06407**

** P<0.01.

Pairwise F_ST_ values ranged from −0.0018 (Taiwan Island and Hainan Island) to 0.4948 (South Asia and Hawaii), and increased with geographic distance. F_ST_ values between Southeast China, Taiwan Island, Hainan Island, Central China and Southeast Asia were low. Relatively high and highly significant, F_ST_ values were found between South Asia or Hawaii and the other five regions (Table 4).

**Table 4:** Pairwise fixation indices (F_ST_) of the seven regions.

Region	1	2	3	4	5	6	7
1. Southeast China	0.0000						
2. Taiwan Island	−0.0083	0.0000					
3. Hainan Island	0.0150	−0.0018	0.0000				
4. Central China	0.0214**	0.0043	0.0026	0.0000			
5. Southeast Asia	0.0603**	0.0316*	0.0227**	0.0316**	0.0000		
6. South Asia	0.1856**	0.1992**	0.1371**	0.1363**	0.1519**	0.0000	
7. Hawaii	0.3836**	0.4735**	0.3853**	0.3275**	0.3330**	0.4948**	0.0000

* P<0.05; **P<0.01.

Effective immigration rates per generation between paired regions were high. No asymmetric immigration rates were found, as indicated by overlapping 95% highest probability density (HPD) intervals between immigration rates into and out of each region. Due to the low effective population size, 0.00372 and 0.00141 in South Asia and Hawaii respectively, gene flows (*Θ*×*M*) entering these two regions were very limited (Table 5).

**Table 5:** Estimates of population size and effective immigration rate between population pairs.

Group	Θ	M						
		1–1→i	2–2→i	3–3→i	4–4→i	5–5→i	6–6→i	7–7→i
1. Southeast China	0.06028	-	447.1 (5.3– 472.0)	301.1 (0.0– 686.7)	564.1 (186.0– 995.3)	190.0 (0.0– 518.0)	359.2 (3.3– 458.0)	377.3 (46.7– 873.3)
2. Taiwan Island	0.05425	499.2 (0.0– 210.0)	-	445.0 (0.0– 896.0)	384.6 (0.0– 808.0)	253.0 (0.0– 623.3)	383.2 (0.0– 697.3)	680.3 (165.3– 1000.0)
3. Hainan Island	0.08198	596.7 (232.0– 994.0)	505.5 (0.0– 566.0)	-	565.8 (206.0– 990.7)	404.5 (62.7– 805.3)	378.3 (0.0– 750.7)	296.0 (0.0– 520.7)
4. Central China	0.09519	748.6 (321.3– 1000.0)	455.7 (18.0– 536.0)	503.1 (4.7– 568.7)	-	423.1 (100.7– 809.3)	226.9 (30.0– 449.3)	102.9 (0.0– 276.7)
5. Southeast Asia	0.06769	313.2 (28.7– 598.7)	156.8 (0.0– 344.7)	193.0 (0.0– 429.3)	437.1 (87.3– 806.7)	-	110.0 (0.0– 360.0)	72.5 (0.0– 211.3)
6. South Asia	0.00372	549.4 (198.7– 998.0)	417.2 (0.0– 886.0)	633.4 (211.3– 1000.0)	455.3 (0.0– 902.0)	245.4 (0.0– 743.3)	-	471.6 (46.0– 946.7)
7. Hawaii	0.00141	274.4 (0.0– 760.0)	291.8 (0.0– 800.7)	253.6 (0.0– 768.0)	198.7 (0.0– 599.3)	183.7 (0.0– 481.3)	373.1 (0.0– 902.7)	-

Θ: mutation-scaled effective population size; M: mutation-scaled effective immigration rate. 95% highest probability density intervals are shown in parentheses. Instances of asymmetrical gene flow are indicated in bold. Source regions are indicated in columns, target regions in rows.

### Phylogeny

**Figure 2:**
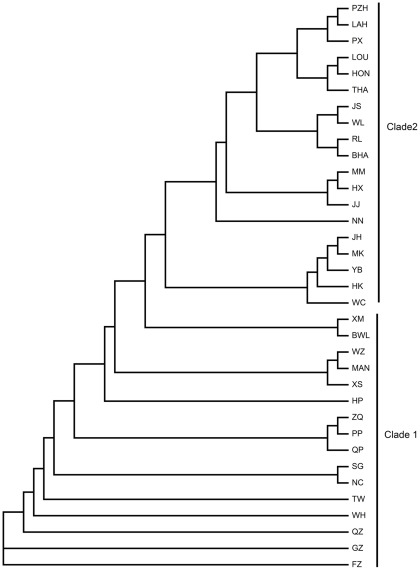
Neighbor-joining tree of the 35 sampled populations. The Taiwan population and almost all of the South-East China populations are situated in the first cluster, almost all of the South Asia populations in the second cluster, and the populations of the Central China region are distributed between both clusters.

The NJ tree of 35 populations (Fig. 2) recovered two clusters. The Taiwan population and almost all of the South-East China populations were situated in the first cluster, almost all of the South Asia populations in the second cluster, and the populations of the Central China region were distributed between both clusters.

**Figure 3:**
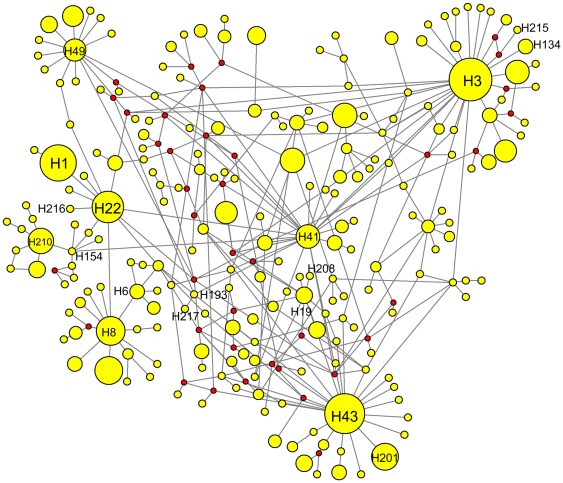
Median-joining network of haplotypes. Node area is proportional to haplotype frequency

A star-like MJ network was constructed, with some high frequency haplotypes (such as H3, H8, H22, H41, H43 and H49) located in the center and other rare haplotypes connected to them through several mutation steps. The haplotypes belonging to the Hawaii region were clearly separated from others in the network, with the exception of H208. H208 connected to H19 through one mutation step, as did the main haplotype of H210 with H154 (Fig. 3). Other haplotypes connected to H210 through several mutation steps (not shown).

### Demographic history

Significantly negative Tajima's D and Fu's *FS* values were found among pooled populations, as well as in the separate populations from Southeast China, Central China, Hainan Island and Southeast Asia, suggesting that the *B. dorsalis* populations of those distribution regions did not conform to the theory of neutral evolution (Table 6).

**Table 6:** Demographic history parameters.

Group	*θ_0_*	*θ_1_*	Τ	Tajima's D	Fu's Fs	SSD
All	0.003	9999.000	4.728	−2.1711**	–24.8351**	0.0021*
Southeast China	0.039	31.826	4.787	−1.6985*	–25.6408**	0.0035
Central China	0.002	9999.000	4.619	–2.1506**	–25.3814**	0.0169*
Hainan Island	0.026	9999.000	4.258	–1.7261*	–25.6720**	0.0886
Taiwan Island	0.002	9999.000	5.828	–0.4168	–1.3513	0.0833
Hawaii	0.001	3.680	2.506	–0.8295	0.2486	0.1346
Southeast Asia	0.021	95.312	4.953	–1.8131**	–25.3379**	0.0008
South Asia	0.001	3.478	4.410	–1.3832	0.0754	0.1488*

*θ_0_*: effective population size before expansion; *θ_1_*: effective population size after expansion; τ: population expansion time; SSD: sum of squared deviations between observed and expected mismatch distributions under a sudden expansion model; *P<0.05; **P<0.01.

**Figure 4:**
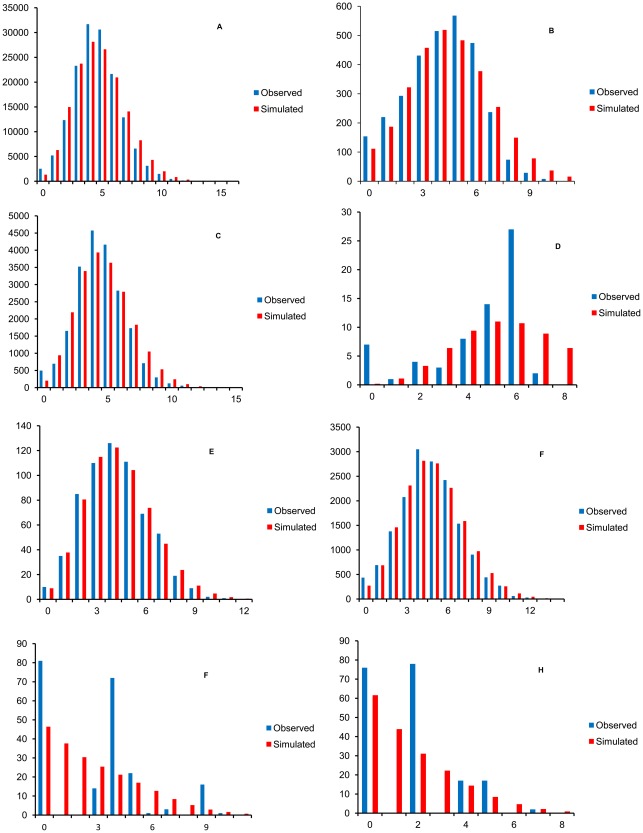
Observed and simulated mismatch distributions. A, all populations; B, South-East China; C, Central China; D, Taiwan Island; E, Hainan Island; F, South-East Asia; G, South Asia; H, Hawaii.

The unimodal mismatch distribution (Fig. 4) of these populations revealed that they were undergoing population expansion; however, a sudden population expansion model was rejected (based on significant SSD values between simulated and observed mismatch distributions) in the case of the pooled populations (P_SSD_ = 0.0032) and the Central China region (P_SSD_ = 0.0169).

Population expansion time (*τ*) in the seven regions ranged from 2.506–5.828. Ratios between the effective population after expansion (*θ_1_*) (3.680–9999.000) and effective population size before expansion (*θ_0_*) (0.001–0.039) implied large population growth.

## Discussion

### Invasion routes

Based on the South-East China origin of the oriental fruit fly [30], [31], the observed increase in F_ST_ values between South-East China and other regions of Asia, together with the increase in the geographic distances, indicate that this species may be colonizing westwards. This is demonstrated by asymmetric gene flow from South-East China to inland [31] and South-East Asia [30]. In the phylogeny of the 35 sampled populations, almost all of the South-East China populations and two South-East Asia populations were found in the first clade of the NJ tree, most South-East Asia populations and one South-East China population in the second clade, and Central China populations distributed among both clades, which suggests that the species may have been colonizing from the endemic region (South-East China) along two independent routes.

One invasion route may run from South-East China to Central China, as indicated by asymmetric gene flows from South-East China to inland China [31]. Sampling records from Mainland China and the genetic data (unimodal mismatch distributions, significant Tajima's *D* and Fu's *FS* values) suggest a gradual invasion process along this route, coupled with rapid population expansion [31]. Many shared haplotypes and high gene flows found between Taiwan Island, Hainan Island and South-East China also imply the same origin region. Although Taiwan Island and Hainan Island are divided from Mainland China by Taiwan Strait and Qiongzhou Strait respectively, the fly would have been able to disperse to the two islands by air and ocean currents [41] and especially via the increasingly frequent fruit and vegetable trade in recent decades [42]. Large numbers of founder individuals may have contributed to the high genetic diversity in the two islands.

Another invasion route may run from South-East China to South East Asia. The fact that the PX population (Guangxi Province, China – part of the South-East Asia region) located on the border of China and Vietnam shared haplotypes with South-East China and other South-East Asia populations (Table S2) indicates that the species may have been invading South-East Asia through Guangxi. Significantly negative Tajima's D and Fu's *FS* values suggest that due to population expansion, selective neutrality is not currently present in this species in South-East Asia [43]. Unimodal mismatch distributions [44] and non-significant SSD values, leading us to reject the null hypothesis of a sudden population expansion model [45], further support this hypothesis. The high genetic diversity of South-East Asian populations found in this study, which has also been detected using nuclear markers [30], indicates an absence of recent genetic bottlenecks. This is likely due to a large number of introduced individuals with high genetic variability, and the abundant host plants and suitable climate in this area. South-East Asia has been suggested as the hypothetical area of originof the oriental fruit fly populations now found in Yunnan, China [46]. Our finding that populations in Yunnan shared haplotypes with several inland China populations such as XS, WZ, JJ, WL and HX (Table S2) suggests that re-invasion into China may have been occurring from Yunnan. A similar invasion route was demonstrated using microsatellite markers [47].

The two independent invasion routes from South-East China may be coinciding in Central China. Populations in this area share haplotypes with both the South-East China and South-East Asia regions and are distributed in both clades of the NJ tree. Genetic admixture supplied by different independent routes in the form of multiple introductions, which have also been found in our previous study [31], is the likely cause of the high genetic diversity of the species in Central China.

Genetic evidence found in this study for the origins of *B. dorsalis* in Hawaii is equivocal. No shared haplotypes were found between Hawaii and other regions. MJ network analysis indicates that haplotype H208, unique to Hawaii, is derived by mutation from H19, mainly found in Taiwan Island, South-East China, Hainan Island and South-East Asia. However the other haplotypes found in this region are mutations of the unique haplotype H154 attributed to Wanzhou, a recently invaded area of Central China. This discrepancy may be due to insufficient haplotype sampling size. Based on historical records and the results of PCR-RFLP analysis of a mitochondrial control region [48], it has been suggested that the oriental fruit fly was introduced to Hawaii from Saipan Island (Northern Mariana Islands) by humans after the Second World War [49]. Although high population densities are found in many areas of the Hawaiian Islands [50], genetic diversity is relatively low (n = 5, H = 0.6000, π = 0.0034, k = 1.7000), which agrees with the results of previous research [30], [48]. Small numbers of initially introduced individuals (*θ_0_* = 0.01) may be the main cause of this genetic homogeneity; constant pest-control pressure associated with strong quarantine regulations imposed on the fruit trade may be another reason. Very limited gene flows detected between Hawaii and other regions indicate that the Pacific Ocean is a barrier to migrational exchange. No shared haplotypes (see also [48]), limited gene flow to other regions and numerous private alleles [30] suggest an independent evolutionary process taking place for *B. dorsalis* populations in the Hawaii Islands.

Introduction to the HP region (India) may have been directly from South-East China, from the nearby region of Myanmar. This interpretation is based on haplotype H6 being shared with the FZ and NN populations, H215, H216 and H217 being mutated from H3 (shared by Taiwan Island and South-East China), H22 (found in South-East China, Central China and South-East Asia) and H193 (unique haplotype of Myanmar). The high genetic diversity of HP (Table 2) is likely due to the different origin sources. Only two haplotypes were found in the LAH population of Pakistan: H134 is shared with the PZH population, and the main haplotype H201 is a mutation of H43 (shared by several Central China and South-East Asia populations). While detailed invasion routes are as yet unknown, it can be inferred that the main area of origin of the LAH population is South-East Asia and that PZH is a secondary source. Although multiple introductions have been observed in LAH, genetic diversity here was very low (Table 2), probably due to a relatively small number of individuals initially introduced to this region.

### Key genetic characters for successful invasion

Because of the considerable differences in ecological conditions between the endemic and non-native regions, natural selection and adaption may be determinants for the success of an invasion before or during the settlement phase. Sufficient genetic variation is essential for evolutionary adaption in response to environmental change and can facilitate the adaption to new environments [24]. High genetic diversity in the oriental fruit fly was observed in its area of origin (South-East China). Large numbers of initially-introduced individuals containing much of the native genetic diversity can be assumed to represent the main contribution to the high genetic diversity in non-native regions like Central China and South-East Asia [51]. Multiple introductions and hybridization among distantly related populations in the non-native range may further enhance genetic diversity. Sufficient genetic variation may facilitate the adaptation of introduced individuals to selection pressures encountered in new habitats during the invasion process and help offset genetic drift in population settle phase with small number of immigrants.

### Management strategies

The oriental fruit fly has to be considered a highly invasive species, as it is able to disperse efficiently and establish adventive populations in various tropical and sub-tropical climate zones. It may however be possible to develop some management strategies based on available invasion history information. For populations that are genetically well-connected and show high gene flow (e.g. Taiwan Island, Hainan Island, Mainland China and South-East Asia), any intervention that is geographically limited to one region is likely to fail, as neighboring populations would readily re-colonize the region. Area-wide interventions aimed at reducing population numbers and economic damage would be a more feasible choice in this case, which would be also appropriate for HON population with relatively low genetic diversity but high population density [50]. For the geographically extreme and genetically independent population LAH, a local intervention aimed at eradication may, however, be the optimal solution.

## Supporting Information

Table S1 Accession numbers of the *cox1* sequences obtained from GenBank.
(DOC)

Table S2 Haplotypes constitution of each population.
(DOC)
