# Supplementary material for: Invasion History of the Oriental Fruit Fly, Bactrocera dorsalis, in the Pacific-Asia Region: Two Main Invasion Routes
Source: PLoS One. 2012 May 2;7(5):e36176. doi: 10.1371/journal.pone.0036176 (PMC3342262; doi:10.1371/journal.pone.0036176)
Supplement: Table S1 — Accession numbers of the cox1 sequences obtained from GenBank. (DOC) [file pone.0036176.s001.doc]

| Population | Accession number |
| --- | --- |
| XM | GQ220668.1-GQ220682.1 |
| QZ | FJ377741.1- FJ377745.1; GQ220650.1-GQ220657.1 |
| ZQ | GQ220683.1-GQ220689.1 |
| MM | FJ377843.1- FJ377847.1 |
| SG | FJ377848.1- FJ377852.1 |
| PX | FJ377783.1- FJ377787.1; GQ220629.1-GQ220636.1 |
| BWL | FJ377836.1- FJ377842.1; GQ220580.1-GQ220588.1 |
| JH | DQ060289-DQ060284 |
| RL | DQ060285; DQ060302-DQ060304; DQ100468; DQ100470; DQ100471; GQ414987 |
| PZH | FJ377788.1-FJ377792.1; GQ220637.1-GQ220642.1 |
| QP | FJ495527.1-FJ495530.1; FJ495532.1; FJ495533.1; FJ495535.1-FJ495544.1 |
| TW | EU076661.1- EU076672.1 |
| YB | DQ060296; DQ060297; DQ060301; GQ414980; GQ414982 |
| MK | DQ060289; DQ060290; DA060295; GQ414975; GQ414977; GQ414978 |
| LOU | FJ377863.1- FJ377872.1 |
| MAN | FJ377853.1- FJ377857.1; GQ220614.1-GQ220622.1; GQ220690.1-GQ220694.1 |
| BHA | DQ060285; DQ060302-DQ060304; DQ100468; DQ100471; GQ414983- GQ414988; |
| THA | EU077539.1-EU077541.1; AY274165.1- AY274168.1; AY053507.1; AY053510.1; AY053512.1 |
| PP | FJ377858.1- FJ377862.1 |
| HP | HQ446514.1- HQ446518.1 |
| HON | FJ377873.1- FJ377875.1; AY945072.1- AY945072.1 |

Table S1 Accession numbers of the *cox1* sequences obtained from GenBank.
