# Supplementary material for: Invasion History of the Oriental Fruit Fly, Bactrocera dorsalis, in the Pacific-Asia Region: Two Main Invasion Routes
Source: PLoS One. 2012 May 2;7(5):e36176. doi: 10.1371/journal.pone.0036176 (PMC3342262; doi:10.1371/journal.pone.0036176)
Supplement: Table S2 — Haplotypes constitution of each population. (DOC) [file pone.0036176.s002.doc]

| Location | Haplotypes |
| --- | --- |
| Fuzhou, China | H1(3), H2(3), H3(5), H4(1), H5(1), H6(1), H7(1), H8(1), H9(1) |
| Xiamen, China | H1(2), H2(1), H3(1), H5(2), H8(1), H10(1), H11(2), H12(1), H13(1), H14(1), H15 (1), H16(1) |
| *Quanzhou, China* | H1(2), H3(4), H4(1), H5(1), H11(1), H13(1), H17(1), H18(1), H19(1) |
| Zhaoqing,China | H3(1), H8(1), H20(1), H21(1), H22(1) , H23(1) , H24(1) |
| Maoming, China | H25(1), H26(1), H27(1), H28(1), H29(1) |
| Shaoguan, China | H1(1), H5(1), H22(1), H30(1), H31(1) |
| Guangzhou, China | H1(1), H3(1), H5(1), H8(4), H20(1), H22(1), H32(1), H33(2), H34(1), H35(1), H36(1), H37(1) |
| Pingxing, China | H13(1), H22(3), H38(1), H39(1), H40(1), H41(1), H42(1), H43(1), H44(1), H45(1), H46(1) |
| Nanning, China | H1(1), H3(1), H6(1), H8(1), H22(1), H43(1), H47(2), H48(1), H49(1), H50(1), H51(1), H52(1), H53(1), H54(1), H55(1), H56(1), H57(1), H58(1), H59(1) |
| Huaxi, China | H3(1), H19(1), H22(1), H60(8), H61(1), H62(1), H63(1), H64(1) |
| Bawangling, China | H5(1), H8(1), H19(1), H22(1), H43(1), H60(1), H65(1), H66(1), H67(1), H68(1), H69(1), H70(1), H71(1), H72(1), H73(1), H74(1) |
| Wenchang, China | H3(2), H5(3), H8(1), H27(1), H41(1), H48(2), H75(1), H76(1), H77(2), H78(1), H79(1), H80(1), H81(1), H82(1), H83(1) |

Table S2. Haplotypes constitution of each population.

| Wuhan, China | H1(2), H3(6), H40(1), H43(3), H84(1), H85(1), H86(1), H87(1), H88(1), H89(1), H90(1), H91(1) |
| --- | --- |
| Nanchang, China | H1(5), H3(2), H8(2), H12(1), H13(2), H16(2), H66(1), H72(1), H92(1), H93(1), H94(1), H95(1) |
| Jianshui, China | H29(1), H43(2), H49(2), H90(1), H96(1), H97(1), H98(1), H99(1), H100(1), H101(1), H102(1), H103(2), H104(1), H105(1), H106(1), H108(1), H109(1) |
| Hekou, China | H8(1), H43(1), H78(1), H110(1), H111(1), H112(1), H113(1), H114(1) |
| Jinghong, China | H113(2), H115(3), H117(4), H118(3), H119(3), H120(1), H121(2), H122(3) |
| Ruili, China | H43(8), H123(5), H124(2), H125(3), H126(1), H127(3), H128(1), H129(2) |
| Panzhihua, China | H22(4), H43(2), H130(1), H131(1), H132(1), H133(1), H134(1), H135(1), H136(1), H137(1) |
| Jiangjin, China | H3(1), H5(2), H8(1), H40(1), H43(2), H60(1), H90(1), H138(1), H139(1), H140(1), H141(1), H142(1), H143(1), H144(1), H145(1), H146(1), H147(1), H148(1) |
| Wanzhou, China | H1(1), H3(3), H6(1), H13(1), H40(1), H47(1), H90(1), H94(1), H114(1), H149(1), H150(1), H151(3), H152(1), H153(1), H154(1), H155(1) |
| Wulong, China | H1(1), H5(1), H22(1), H23(1), H36(2), H43(1), H48(1), H49(1), H50(1) H106(1), H156(1), H157(1), H158(1), H159(1), H160(1), H161(1), H162(1), H163(1), H164(1) |
| Xiushan, China | H3(2), H8(1), H12(1), H16(1), H18(2), H22(1), H45(1), H47(1), H48(1), H165(2), H166(1), H167(1), H168(2), H169(1), H170(1), H171(1) |
| *Qingpu, China* | H1(5), H3(5), H4(2), H7(2), H13(1), H18(1) |

Table S2 Cont.

| *Taiwan, China* | H3(4) , H5(1), H13(2), H16(1), H19(1), H172(1), H173(1), H174(1) |
| --- | --- |
| Yei Bai,Vietnam | H43(3), H175(4), H176(2), H177(3), H178(9) |
| Muang Khu, Laos | H115(6), H116(8), H122(3), H179(2), H180(1) |
| Louangphabang, Laos | H19(1), H43(1), H68(1), H181(1), H182(1), H183(1), H184(1), H185(1), H186(1), H187(1) |
| Mandalay, Myanmar | H3(2), H22(1), H41(1), H43(1), H49(1), H63(1), H67(1), H109(2), H188(1), H189(1), H190(1), H191(1), H192(1), H193(1), H194(1), H195(1), H196(1) |
| Bhamo, Myanmar | H41(7), H43(2), H124(1), H125(1), H126(1), H127(1), H129(9), H197(2), H198(2), H199(1), H200(1) |
| *Thailand* | H22(2), H48(1), H68(1), H202(1), H203(1), H204(1), H205(1), H206(1), H207(1) |
| *Phom Penh, Cambodia* | H23(2), H112(1), H213(1), H214(1) |
| Lahore, Pakistan | H134(3), H201(13) |
| Himachal Pradesh, India | H6(1), H33(1), H215(1), H216(1), H217(1) |
| *Honolulu , USA* | H208(1), H209(5), H210(12), H211(1), H212(1) |

Table S2 Cont.
